# The glass transition of biologically secreted amorphous calcium carbonate

**DOI:** 10.1039/d6ma00224b

**Published:** 2026-07-07

**Authors:** Thilo Bissbort, Kai-Uwe Hess, Erika Griesshaber, Carmen Salas, Antonio Checa, Wolfgang Schmahl, Donald B. Dingwell

**Affiliations:** a Earth and Environmental Sciences, Ludwig-Maximilians-Universität München Theresienstraße 41/III 80333 München Germany thilo.bissbort@ruhr-uni-bochum.de; b Department of Animal Biology, Universidad de Málaga Málaga Spain; c Departmento de Estratigrafia y Paleontología, University of Granada Spain; d GEOLAB, Hangzhou International Innovation Institute 311115 China

## Abstract

Amorphous carbonates are key phases in bio-geological processes. They also possess considerable unexplored potential for technological applications. We recovered a glass transition in biogenic amorphous calcium carbonate (ACC), extracted from the soft tissue of the sea slug *Baptodoris cinnabarina*, using novel fast differential scanning calorimetry (FDSC) (heating/cooling rates of 2000–5000 °C s^−1^). Similar to synthetic ACC and ACMC, biogenic ACC, as demonstrated here, is a structural glass. Thus, synthetic and biogenic ACC likely share the same vitrification route – dehydration-driven glass formation. Experimental strategies employed here reveal that biogenic ACC exhibits an enhanced stability compared to synthetic ACC. This is interpreted as a crystallization-inhibiting role of Mg, P, and organics, initially present in biogenic ACC as has been previously inferred.

## Abstract 2

The sea slug *Baptodoris cinnabarina* secretes amorphous calcium carbonate (ACC) in the form of a structural glass, which is more stable than synthetic ACC. It is likely produced through dehydration-driven glass formation, similar to synthetic ACC.

## Graphical abstract

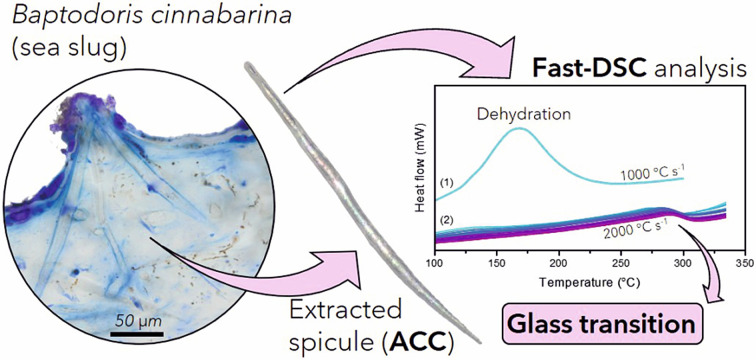

Amorphous carbonates have gained significant attention in recent decades. This has been primarily a consequence of their relevance in natural and technological sciences. Firstly, amorphous carbonates are being increasingly identified to play crucial roles in different substances and processes that cover various scientific disciplines like material science, chemistry, marine biology and geoscience. Solid amorphous carbonates, in particular amorphous calcium carbonate (ACC) and amorphous calcium-magnesium carbonates (ACMC), have been recognized as key phases in biomineralization,^1–6^ where ACC represents the least stable polymorph in a transformation sequence involving vaterite, aragonite, and finally calcite.^2^ Secondly, the exceptional properties of amorphous carbonates make them attractive materials for technological applications, for example in food sciences and pharmaceutics.^7–9^ As the significant relevance of amorphous carbonates becomes increasingly clear, investigation of their properties remains a major challenge. Although manifold studies have utilized a variety of analytical techniques (*e.g.*, NMR,^10^ TEM,^11^ FTIR,^12^ XRD^13^), computer simulations,^14–16^ and sample types (biogenic and synthetic ACC and ACMC), an adequate understanding of the formation mechanisms and the properties of ACC and ACMC remains elusive.

Currently, synthetic ACC and ACMC are routinely synthesized by precipitation-lyophilization methods.^17,18^ However, the relatively fragile state of ACC and ACMC under laboratory conditions (*i.e.*, alteration within hours during storage) and their tendency towards modification during thermal analysis (*e.g.*, beam damage in TEM analyses or rapid crystallization and dehydration in calorimetric measurements^11^) render their investigation challenging. Recently, we examined synthetic ACC and ACMC in experiments that involved novel fast differential scanning calorimetry (FDSC). We were able to study the behaviour of nominally anhydrous synthetic ACC and ACMC during heat treatment at extremely high heating and cooling rates (500–6000 °C s^−1^).^11,19^ The obtained heat flow curves exhibited the clear endothermic signals of the glass transition. Thus, anhydrous ACC and ACMC clearly behave as structural glasses.^11,19^ This is somewhat remarkable in light of the fact that these glasses are not produced by the typical thermal route of quenching from a melt but rather, as noted above, by precipitation-lyophilization. A subsequent parallel study on hydrous synthetic ACC and ACMC with variable water contents revealed a strong effect of water content on the glass transition temperature, consistent with the notion that synthetic ACC and ACMC can be formed isothermally by crossing the glass transition during dehydration, thereby vitrifying from the aqueous solution.^20^

Amongst other applications, synthetic ACC and ACMC can be viewed, in terms of both composition and formation mechanism, as simplified analogues of naturally occurring biogenic amorphous calcium carbonate, whose formation mechanism and properties are poorly constrained. It has been demonstrated previously that natural ACC, as it is observed, for example, in sea urchins,^3,4,21–23^ can contain phosphorus,^24–26^ proteins^4^ and up to 5 mol% Mg (ACMC),^4^ apparently enhancing the stability of the amorphous phase. It remains unclear whether ACC formed by organisms is (i) a structural glass and (ii) more stable than synthetic ACC. Clearly, answers to these questions would bear direct implications for its biogenic formation.

Here, we perform FDSC analysis on biogenic ACC, namely on the spicules of the sea slug *Baptodoris cinnabarina* (Discodorididae, Doridida) (Fig. 1). The spicules are about 200 to 400 µm long and 20 to 30 µm wide. Recently, the state of the Ca-carbonate spicules of the sea slug was characterised using a multi-analytical approach.^27^ In that study, micro-CT analysis revealed that this organism contains, as support of its soft tissue, a 3D network of numerous spicules within the integument of the dorsal body-surface. SEM and TEM analysis showed that individual spicules have a rim with a concentrically layered structure, made of organic laminae and intercalations of ACC granules. The inner core structurally differs from the rim due the absence of layering, with the organic material being restricted to fillings of locally isolated interstitial spaces. Cracks that originate in the core of the spicules are terminated at the core-rim interface, where organic films are present, potentially indicating a mechanically stabilizing role of the organic material. SAED patterns and XRD analysis confirmed that all the mineralized substance is amorphous. ^13^C CP MAS NMR and ^31^P solid state NMR analysis was used to detect small amounts of organics, ACC, and amorphous calcium phosphate (ACP). Further, 2D ^1^H–^31^P NMR measurements indicate the presence of water molecules that are associated with the phosphate of the ACP. Element distribution maps and single point analysis performed with EDS show that spicule rims have high P and Mg concentrations, while core areas have low P and variable Mg contents.

**Fig. 1:**
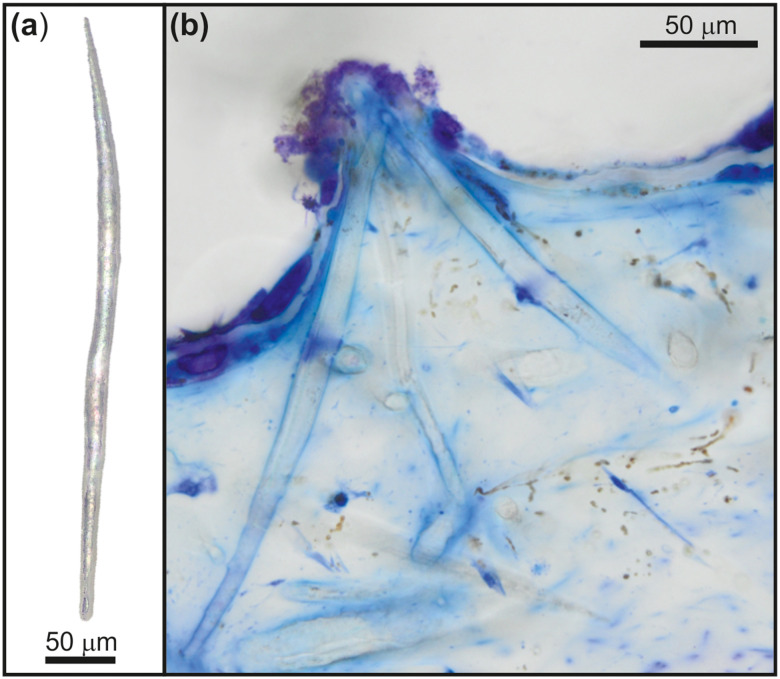
(a) Optical image of a single spicule from *Baptodoris cinnabarina*. (b) Optical view of a transversal section of one caryophyllid (a dorsal tubercle of the mantle supported by internal spicules) from a methacrylate-embedded specimen.

Here, these spicules were mechanically processed to obtain fragments, from which particles of the adequate size for FDSC analysis were selected.

The Flash DSC 2+ (Mettler-Toledo) was used with a UFH1 calorimeter chip. Heating and cooling rates between 2000 and 5000 °C s^−1^ were applied to prevent crystallization while minimizing effects of thermal inertia. The sample side of the sensors were coated with a thin layer of silicon oil (AK 500.000 Wacker) to improve the thermal contact between sensor and samples possessing an irregular shape.^28^ All FSDC measurements were performed in a CO_2_ flow of 30 mL min^−1^. The glass transition temperature is defined as the “limiting fictive temperature”^29^ (*T*_f_). This definition quantifies *T*_f_ by the integral technique. All samples were intentionally heated until crystallized after successful completion of the analyses, to obtain heat flow curves that were used for baseline corrections. Characteristic temperatures were determined solely from the heat flow curves of samples that had undergone no crystallization. The onset of melting of Sn, Bi, Al and the alpha-beta quartz transition were used to apply a linear temperature correction to all measurements (see SI for details).

We applied three different experimental strategies using FDSC to explore the behaviour of biogenic ACC during heating and cooling, thereby taking full advantage of the extremely high heating and cooling rates of FDSC.

(i) The aim of the first type of analysis was to understand the response of a pristine sample to FDSC measurements. Biogenic ACC was heated without any thermal pre-treatment from 30 °C to 800 °C at 2000 °C s^−1^. The resulting heat flow curve is displayed as the blue line in Fig. 2. A first strong, broad, and endothermic signal was observed between *ca.* 100 and 250 °C. The near-Gaussian-shaped signal and its wide temperature range are typical for dehydration events. Similar signals have been observed in our previous studies of synthetic hydrous ACC and ACMC.^20^ Further heating leads to a section of the curve that starts at about 360 °C and which ends at *ca.* 420 °C. The heat flow curves in this temperature range consist of several intense, more discrete endothermic (decarbonatization and thermal decomposition) and some smaller exothermic (crystallization) peaks.

**Fig. 2:**
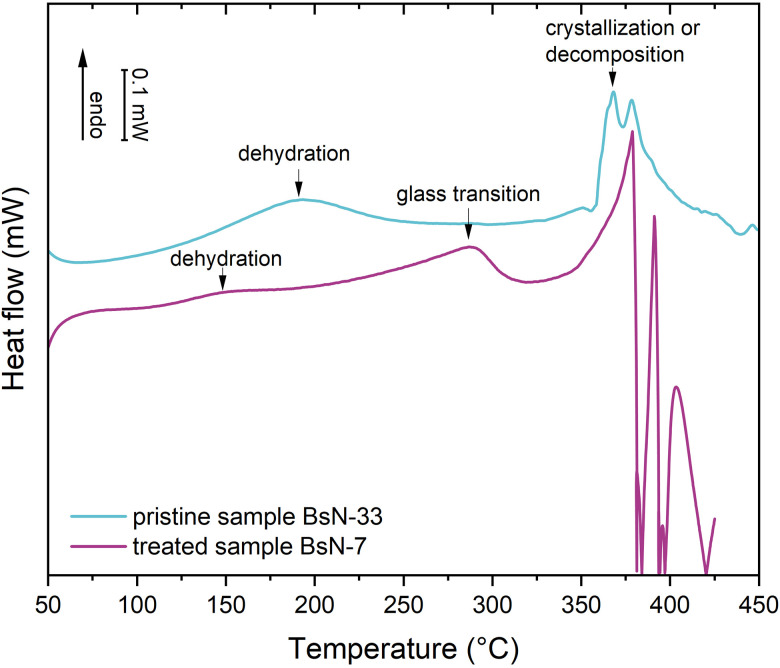
FDSC heat flow curves obtained using analytical strategy (i) for an untreated ACC sample (BsN-33, blue) and, for comparison, an analysis of a dehydrated ACC sample (BsN-7, purple) thermally pretreated following strategy (iii). The part between 450 °C and 800 °C for BsN-33 is not shown here for improved clarity and is irrelevant for the aim of this study, see text.

At higher temperatures the signals are overall less intense and are endothermic and exothermic (not shown here). No endothermic signal, which could be interpreted as a glass transition, was observed in this measurement approach and the crystallization and thermal decomposition at elevated temperatures left no glassy material to be analysed. It is therefore clear that a major challenge is the dehydration signal which potentially masks a signal related to the glass transition, while at higher temperatures the investigated material is unstable.

(ii) The repeatedly heating of a pristine sample to incrementally increasing maximum temperatures up to a final temperature of 350 °C at heating and cooling rates of 2000 °C s^−1^ allows a deeper probing of the sample behaviour during heating and cooling. This type of analysis has recently been successfully applied to explore the glass transition region of extremely fragile glasses, such as highly depolymerized silicate glasses.^30^ Heat flow curves obtained from these successive heating events are shown in Fig. 3. Similarly to measurement (i), the heat flow increases in the temperature region around 100 °C. However, with successive cycles, the signal flattens until the heat flow curves converge in this temperature range. Instead, another endothermic signal at higher temperatures is uncovered by the continuous dehydration of the sample. This signal shifts slightly towards higher temperatures with successive heating events until it attains a final state, *i.e.*, constant temperature and shape, unchanged by further cycles. The sequence of heating events has led to a dehydration of the sample, which is a modification of the sample composition, manifested in the occurrence of a dehydration signal which progressively vanishes with sustained heating. The higher temperature endothermic signal initially shifts towards higher temperatures with ongoing dehydration. Such an increase in glass transition temperatures with decreasing water content has been well-established for synthetic ACC and ACMC.^20^ This relationship, which is clearly reflected in our measurements, together with the final reproducibility of the endothermic signal in terms of shape, amplitude, and temperature enables us to rule out the possibility that it is related to another process involving a change in composition, (*e.g.*, another dehydration event) implying that it is, in fact, the glass transition. Further, the reproducibility associated with the glass transition implies that a constant degree of dehydration has been achieved and that no further modification of the sample is occurring.

**Fig. 3:**
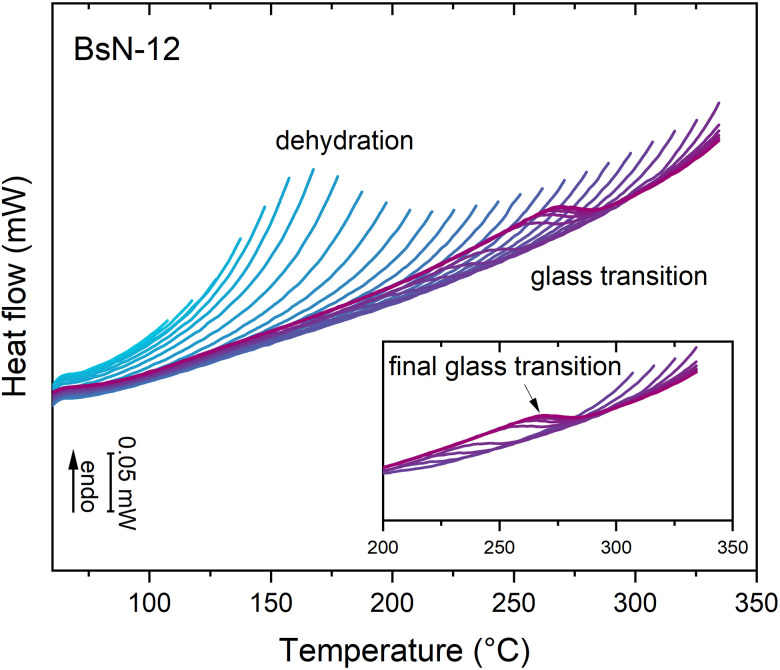
Heat flow curves obtained in FDSC measurements (ii) of biogenic ACC (BsN-12) at heating and cooling rates of 2000 °C s^−1^. The colour gradient from blue to purple indicates the chronological order of measurements with incrementally increasing maximum temperatures, bright blue being the first and purple the final measurement. The inset shows magnification of the glass transition signal.

Attempts to dehydrate the material through isothermal holds at lower temperatures, for example at 150 °C, as it has been done for the synthetic material, were unsuccessful, apparently reflecting a very inefficient dehydration at low temperatures, such that the material exhibited a progressively decreasing dehydration signal in successive measurements.

Thus, another experimental strategy (iii) involving an initial heating of samples to 300 °C at 1000 °C s^−1^, far from the onset of crystallization and thermal decomposition but high enough in temperature to allow almost complete dehydration, confirmed by the observation that almost no further dehydration effects were observed in subsequent heat flow curves (Fig. 4a), was applied. The following heat flow curve at 2000 °C s^−1^ to 350 °C exhibits merely a minor dehydration signal which vanishes during the next heating–cooling cycles. Due to the initial near-complete dehydration, the endothermic glass transition signal is already visible in the second heating event. It shifts towards higher temperatures as final dehydration is approached and, similarly to results from analysis strategy (ii), attains a constant and reproducible form in all subsequent measurements (Fig. 4b). Finally, the glass transition temperature, which we define here as the limiting fictive temperature^29^ (*T*_f_), is a function of the cooling rate. Hence, higher cooling rates will lead to a crossing of the glass transition at higher temperatures, *i.e.*, higher *T*_f_. We have quantified this by applying matching heating–cooling measurements at different rates between 2000 and 5000 °C s^−1^. Five heating–cooling cycles were performed at a single rate, the first one was discarded because its glass transition signal is related to the previous non-matching rate, and the remaining four heat flow curves were averaged after assessing their consistency. *T*_f_ and the peak temperatures of the signals (*T*_peak_) were determined from these final heat flow curves and are listed in SI. The difference in the temperature range of the glass transition as a function of cooling rate was already evident in a first test using 2000 °C s^−1^ and the maximum 5000 °C s^−1^ (Fig. 4a). A further nominally anhydrous sample was used to study this aspect at several matching heat-cooling rates between 2000 and 5000 °C s^−1^ (Fig. 4c). The temperature range of the glass transition and the associated characteristic temperatures *T*_f_ and *T*_peak_ clearly increases with increasing rates. This is further evidence that the observed endothermic signal is a glass transition. The relationship is described by an Arrhenian law with activation energies of 102 ± 11 and 117 ± 3 kJ mol^−1^, extracted from measurements of two samples (see SI). However, we note that these values are representative for the specific samples measured, due to the small sample size that is probed and the structural and compositional variability within individual spicules (see above and Griesshaber *et al.*^27^).

**Fig. 4:**
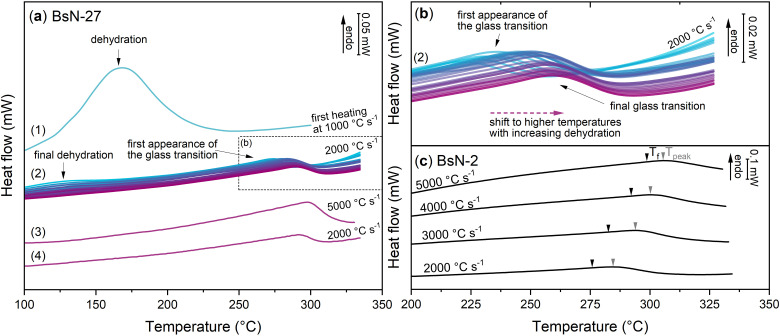
(a) Overview of heat flow curves obtained from the experimental strategy (iii). The numbers indicate the chronological order of analyses. Initial heating at 1000 °C s^−1^ to 300 °C induces dehydration of the sample BsN-27 (1). Following heating events at 2000 °C s^−1^ up to 350 °C finalize the dehydration process leading to a constant, reproducible glass transition signal (2). A magnification of this process is shown in panel (b). (2), (3) and (4) show a comparison of the glass transition region using different matching heating- and cooling rates. (c) Shows a systematic increase in the glass transition temperature range with increasing cooling rates for sample BsN-2, which was previously dehydrated, like sample BsN-27, following strategy (iii). *T*_f_: limiting fictive temperature; *T*_peak_: peak temperature.

A return to 2000 °C s^−1^ in a final analysis after measurements at higher rates to test for any instrument or sample drift reproduces well the glass transition signal of previous measurements at the same rate, in terms of signal intensity and associated temperature (*cf.*, heat flow curves (2), (3) and (4) in Fig. 4a). This confirms that the sample did not undergo modification, *e.g.*, crystallization or further dehydration, during measurements which could have altered the glass transition signal. Rather, the shift in temperature is a result of the cooling rate dependence of the limiting fictive temperature. Results from a numerical heat-flow model (see SI) confirm that thermal inertia at heating rates (max. 5000 °C s^−1^) applied here and the used sample size (∅ < 30 µm) is insignificant and does not affect the results. *T*_f_ and *T*_peak_ values at 2000 °C s^−1^ were quantified for eleven samples in total (see SI). *T*_f_ ranges from 233 to 283 °C and *T*_peak_ from 253 to 296 °C, with the temperature intervals reflecting the origin of samples from different spicule segments and therefore inferred compositional differences.

In conclusion, we applied different analytical strategies using FDSC at extremely high heating–cooling rates (2000–5000 °C s^−1^) to separate and observe, dehydration, the glass transition, and crystallization of biogenic ACC from spicules of the sea slug *Baptodoris cinnabarina*.

Our investigation demonstrates that biogenic ACC, as it is found in *Baptodoris cinnabarina*, is a structural glass. A glass transition in this material is confirmed by (1) the reproducibility of the associated endothermic signal in numerous subsequent heating–cooling cycles, (2) the increase in glass transition temperatures with progressive dehydration, consistent with previous findings for the synthetic analogue, and (3) the rate-dependence of *T*_f_. This is particularly significant because the glass was not formed by thermal quenching from a melt but was biologically secreted. Instead, the fact that biogenic ACC exhibits a glass transition like synthetic ACC and ACMC, which are synthesized by lyophilization,^18,20^ indicates that it could be produced by a route similar to that proposed for the synthetic analogues, *i.e.*, dehydration-driven glass formation.^20^ Hence, biogenic ACC may form by crossing the glass transition isothermally from a dense ion-rich liquid precursor phase to a solid glassy ACC by dehydration as previously described.^20^

The nature of the initial liquid(-like) phase, which undergoes dehydration to form solid ACC, is a subject of ongoing debate.^31–34^ Experiments and molecular dynamics simulations in the CaCO_3_–H_2_O system suggest that this precursor is a dense ion-rich liquid that was produced by liquid–liquid separation in a supersaturated aqueous medium.^31,34^ Experimental studies that analysed phase transformations in a CaCO_3_–H_2_O system that contains polymers such as poly-aspartic acid (pAsp) indicate that these charged additives form a polymer-induced liquid precursor (PILP) which contains polymer-stabilized ACC nanoclusters, while no liquid–liquid separation was detected.^32,33^

Our results show that ACC forms as a structural glass in complex biological systems involving, Mg, P, and organics but also under controlled laboratory conditions in relatively simple systems in the absence of organics like pAsp.^20^ The presence of polymers during the formation of glassy ACC is therefore not necessarily required but its stability-enhancing effect might be reflected in the different behaviour of the two types of ACC in our thermal analyses. The feasibility of numerous excursions across the glass transition without crystallization using biogenic ACC, which is demonstrated by the absence of exothermic signals and the large temperature interval between glass transition and the onset of crystallization compared to synthetic ACC, *e.g.* 60 *versus* 5 °C at 2000 °C s^−1^ respectively, together with the reproducibility of glass transition signals, is consistent with the assertion that the biogenic ACC studied here is much more stable than the synthetic analogue. Synthetic ACC and ACMC typically crystallize upon crossing the glass transition.^11,19,20^ This contrasting behaviour is in agreement with observations made by others that phosphate,^25,26,35^ magnesium,^4,26,36^ and proteins^3,4,25^ yield a stabilizing effect, inhibiting or delaying a transformation to crystalline phases.

Future work should determine the effect of single components such as phosphate or proteins on the glass transition. This might shed light on why spicules exhibit layers and sections (cores and rims) with different structures and compositions. Another further open question that should be addressed in future studies is that of the mechanism by which ACC-bearing organisms facilitate the dehydration process, which is fundamental to the formation of the glass.

## Author contributions

TB: conceptualization, data curation, formal analysis, investigation, validation, visualization, writing – original draft, writing – review & editing; KUH: conceptualization, validation, writing – original draft, writing – review & editing; EG: resources, writing – original draft, writing – review & editing; CS: resources, writing – review & editing; AGC: resources, writing – review & editing; WS: resources, writing – original draft, writing – review & editing; DBD: funding acquisition, resources, writing – original draft, writing – review & editing.

## Conflicts of interest

There are no conflicts to declare.

## Supplementary Material

MA-007-D6MA00224B-s001

## Data Availability

The data supporting this article have been included as part of the supplementary information (SI). Supplementary information is available. See DOI: https://doi.org/10.1039/d6ma00224b. FDSC measurement data are available at Zenodo at https://doi.org/10.5281/zenodo.18661158.
