# Supplementary material for: The glass transition of biologically secreted amorphous calcium carbonate
Source: Mater Adv. 2026 Jul 7;7(15):7558–63. doi: 10.1039/d6ma00224b (PMC13360893; doi:10.1039/d6ma00224b)
Supplement: MA-007-D6MA00224B-s001 [file MA-007-D6MA00224B-s001.pdf]

Supplementary Information (SI) for Materials Advances.
This journal is © The Royal Society of Chemistry 2026

Supporting Information for:

The glass transition of biologically secreted amorphous calcium carbonate

Thilo Bissbort,*[a,1] Kai-Uwe Hess,[a] Erika Griesshaber,[a] C. Salas,[b] A. Checa,[c] Wolfgang Schmahl[a] and Donald B. Dingwell[a,d]

[a] *Earth and Environmental Sciences, Ludwig-Maximilians-Universität München, Theresienstraße 41/III, 80333 München, Germany.*
[b] *Department of Animal Biology, Universidad de Málaga, Málaga, Spain*
[c] *Departmento de Estratigrafia y Paleontología, University of Granada, Spain*
[d] *GEOLAB, Hangzhou International Innovation Institute, 311115, China*
[1] *Present address: Institute for Geology, Mineralogy and Geophysics, Ruhr-Universität Bochum, 44780 Bochum, Germany*
[+] *Corresponding author*

Content:

S1: Limiting fictive temperatures and peak temperatures of biogenic ACC

- Figure S1: T_f and T_{peak} of eleven biogenic ACC samples
- Figure S2: T_f as a function of cooling rate and activation energies
- Table S1: T_f and T_{peak} of eleven biogenic ACC samples
- Table S2: Cooling-rate dependence of T_f and T_{peak}

S2: Evaluation of thermal lag in FDSC analysis and temperature calibration

- Figure S2: Model result for a thermal profile resulting from FDSC analysis
- Table S3: Temperature calibration of UFH1 sensors

References

S1: Limiting fictive temperatures and peak temperatures of biogenic ACC

The limiting fictive temperatures and peak temperatures of eleven samples of biogenic ACC obtained from spicules of the sea slug *Baptodoris cinnabarina* were quantified in FDSC measurements at matching heating-cooling rates of 2000 °C s^{-1}. The peak temperature is the temperature at which the peak of the endothermal glass transition signal appears. The limiting fictive temperature is the glass transition temperature defined after Moynihan et al.[1] and Richardson[2] and was determined using the evaluation software of Mettler-Toledo using the feature "glass transition temperature according to Richardson". Temperatures were quantified only in heat flow curves that were reproduced in consecutive cycles, which implies that no further dehydration took place and that the samples were not modified otherwise, e.g., by crystallization. Samples BsN-1 to BsN-11 and BsN-27 were measured following the analytical strategy (iii) and samples BsN-12 to BsN-14 using strategy (ii) (Figure S 1). The temperature values are listed in Table S 1. T_f ranges from 233 to 283 °C and T_{peak} from 253 to 296 °C. The temperature range is caused by the origin of samples from different parts of the spicules. Two samples BsN-1 and BsN-2 were dehydrated following strategy (iii) and were used to determine the cooling-rate dependence of the characteristic temperatures (Table S 2 and Figure S 2).

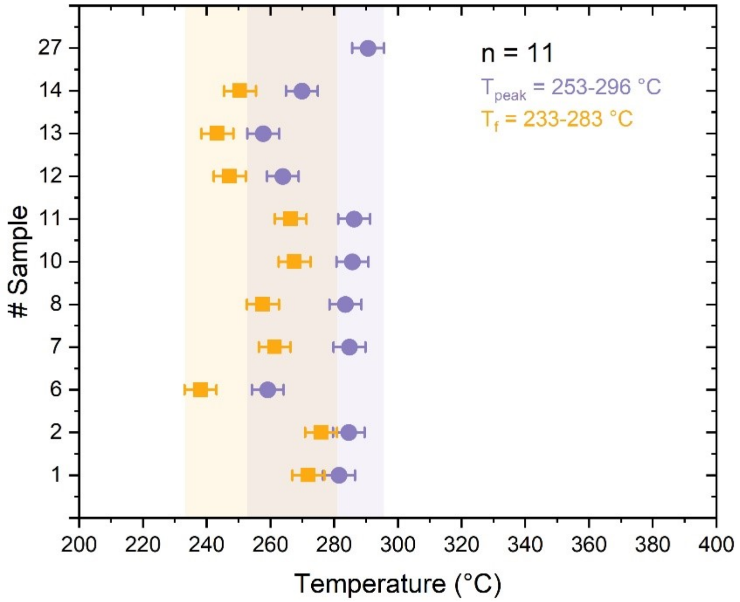

Figure S 1: A total of 11 samples of biogenic ACC was measured at 2000 °C s^{-1} using the analytical strategy (iii) (samples 1-11, 27) and strategy (ii) (samples 12-14), see main text for analytical details. Limiting fictive temperatures (T_f) are indicated by yellow squares and peak temperatures (T_{peak}) by purple circles. The two vertical bands are the temperature ranges covered by the eleven samples.

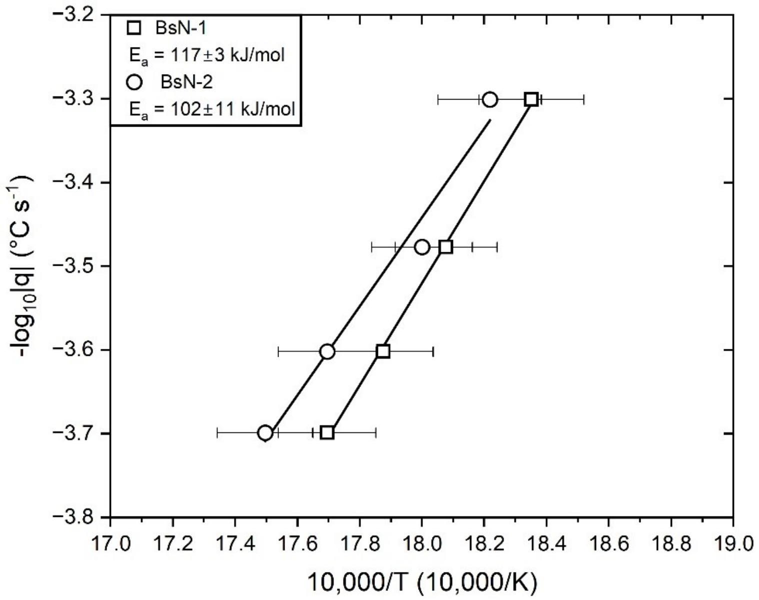

Figure S 2: The fictive limiting temperatures for two samples were measured at different cooling rates (2000-5000 °C $^{-1}$). These show an increase with increasing cooling rate, typical for the fictive limiting temperature. Corresponding values are reported in Table S 2.

Table S 1: Limiting fictive temperatures (T_f) and peak temperatures (T_{peak}) determined at matching heating-cooling rates of 2000 °C s^{-1} for eleven biogenic ACC samples. The uncertainty in obtained temperatures is ±5 °C.

# Sample	T_f (°C)	T_{peak} (°C)	Analytical strategy
BsN-1	272	282	iii
BsN-2	276	285	iii
BsN-6	238	259	iii
BsN-7	261	285	iii
BsN-8	258	284	iii
BsN-10	268	286	iii
BsN-11	266	286	iii
BsN-12	247	264	ii
BsN-13	243	258	ii
BsN-14	250	270	ii
BsN-27	278	291	iii

Table S 2: Limiting fictive temperatures (T_f) and peak temperatures (T_{peak}) determined at different matching heating-cooling rates between 2000 and 5000 °C s^{-1} for two biogenic ACC samples. The samples were previously dehydrated following analytical strategy (iii). The uncertainty in obtained temperatures is ±5 °C.

# Sample	Heating-cooling rate (°C s^{-1})	T_f (°C)	T_{peak} (°C)
BsN-1	2000	272	282
	3000	280	289

	4000	286	294
	5000	292	300
BsN-2	2000	276	285
	3000	283	294
	4000	292	300
	5000	299	305

S2: Evaluation of thermal lag in FDSC analysis and temperature calibration

We have employed a thermal conduction model to evaluate potential thermal inertia during heating of ACC samples in FDSC analysis. Fourier's law of heat conduction (eq. S2.1) was solved numerically using a one-dimensional finite difference scheme, where T is the temperature in K, t is the time in s, x is the distance in m, and α is the thermal diffusivity in m² s^{-1}. Thermal diffusivity α is a function of the thermal conductivity k in W m^{-1} K^{-1}, the density ρ in kg m^{-3}, and the heat capacity c_p of the material in J kg^{-1} K^{-1} (equation S2.2).

$$\left.\frac{\partial T}{\partial t}\right|_x = \alpha \cdot \frac{\partial^2 T}{\partial x^2} \qquad \text{(equation S2.1)}$$

$$\alpha = \frac{k}{\rho \cdot c_p} \qquad \text{(equation S2.2)}$$

We have used a thermal conductivity of 3.2 W m^{-1} K^{-1}, which is the thermal conductivity of calcite[3], a density of 2710 kg m^{-3} for ACC[4], and a heat capacity of 1120 J kg^{-1} K^{-1} calculated for calcite at 350 °C[5]. We have deliberately chosen variable values represent the worst-case scenario, hence most intense thermal lag. The thermal diffusivity was held constant in our calculations. The sample thickness was 25 µm, which was the upper limit of sample dimension in this study. The model uses a starting temperature of 30 °C and a final temperature of 350 °C, the relevant temperature range in the context of this work. The effect of different heating rates between 2000 – 5000 °C s^{-1} was calculated. A Dirichlet condition on the sensor – sample contact induces an increase in temperature over time. A Neumann condition on the other boundary imposed a closed boundary condition. The deviation in temperature from the imposed temperature, which is representative for the thermal lag, with increasing distance to the sensor–sample interface, is extremely small (Figure S 3). The maximum difference (ΔT_{max}) calculated for the far end of the sample was -1.48 °C from the final temperature of 350 °C at the maximum heating rate of 5000 °C s^{-1}. At the lowest heating rate of 2000 °C s^{-1} ΔT_{max} is -0.59 °C. These temperature differences are negligible and below the uncertainty of FDSC analysis of ± 3°C and well below the changes in fictive limiting temperature T_f with increasing cooling rate (for example for sample BsN2 ΔTf = 23 °C between 2000 and 5000 °C s^{-1}).

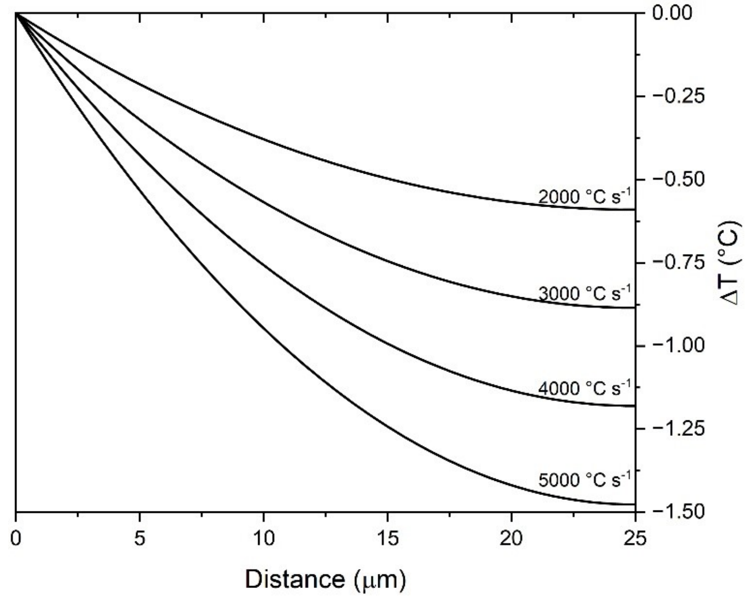

Figure S 3: Temperature difference ΔT between the temperature at the sensor – sample contact (0 μm) and the temperature as function of distance at the final temperature of 350 °C after heating a sample of 25 μm thickness from 30 to 350 °C at different rates applied in this study.

Temperature calibrations for UFH1 sensors were performed using the onset of melting of Sn, Bi, Al and the onset of the alpha-beta transition in quartz (Table S 3 and Figure S 4). Small pieces of calibration substance were placed on the reference sides of sensors. The calibration protocol consists of heating from 30 °C to a temperature above the expected temperature of the phase transition, e.g., +50 °C, at 1000 °C s^{-1}, followed by subsequent cooling at 1000 °C s^{-1} to 30 °C. The determined onset temperatures are reported in Table S 3. Very small sample sizes can be used for calibration due to the intense peaks associated with the phase transitions. Additional measurements at faster (5,000 °C s^{-1}) and slower rates (50 °C s^{-1}) indicate that the onset temperatures are insensitive to heating rates and are therefore unaffected by thermal lag. This agrees with results from the original calibration protocol proposed for these kind of sensors by Zhuravlev and Schick (2010)[6]. The difference between the measured onset temperature of melting of Al and the reference temperature (660.3 °C), here 43 °C, is comparable to that determined by Schawe and Hess (2019)[7], which is 39 °C. A simultaneous assessment of thermal lag and temperature offset by placing the reference material on top of the sample, as done by others[6,8] was not possible due to the irregular shape of ACC samples. The collection of measured onset temperatures of the different calibrants was used to obtain a linear temperature correction.

Table S 3: Temperature calibration of UFH1 sensors

Reference material	Transition	Reference temperature (°C)	Measured onset temperature	ΔT (°C)
Sn	Melting	231.9	236	4
Bi	Melting	271.4	276	5
Al	Melting	660.3	703	43
Qtz	Alpha-beta	573	614	41

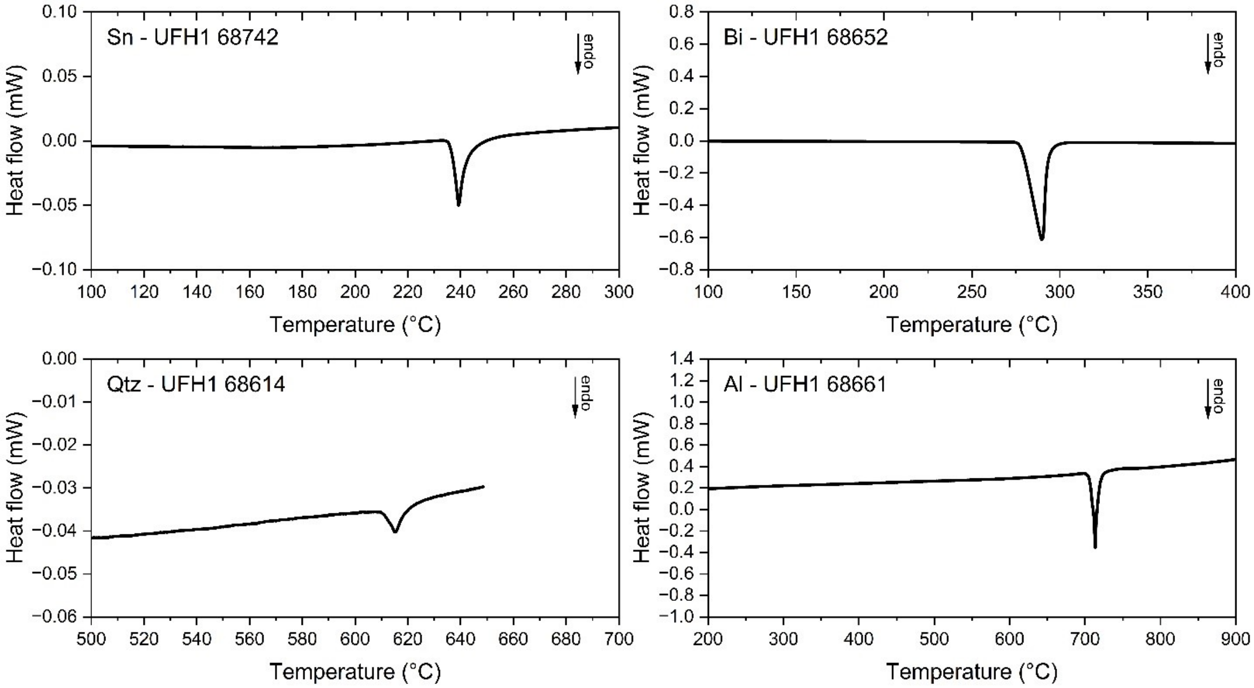

Figure S 4: A selection of representative heat flow curves of Sn, Bi, Qtz, and Al used for temperature correction of UFH1 sensors.

References

1 C. T. Moynihan, A. J. Easteal, M. A. DeBolt and J. Tucker, Dependence of the fictive temperature of glass on cooling rate, *J American Ceramic Society*, 1976, **59**, 12–16.

2 M. J. Richardson and N. G. Savill, Derivation of accurate glass transition temperatures by differential scanning calorimetry, *Polymer*, 1975, **16**, 753–757.

3 J. Thomas, R. R. Frost and R. D. Harvey, Thermal conductivity of carbonate rocks, *Engineering Geology*, 1973, **7**, 3–12.

4 M. Saharay, A. O. Yazaydin and R. J. Kirkpatrick, Dehydration-induced amorphous phases of calcium carbonate, *The journal of physical chemistry. B*, 2013, **117**, 3328–3336.

5 G. K. Jacobs, D. M. Kerrick and K. M. Krupka, The high-temperature heat capacity of natural calcite (CaCO3), *Phys Chem Min*, 1981, **7**, 55–59.
6 E. Zhuravlev and C. Schick, Fast scanning power compensated differential scanning nano-calorimeter: 2. Heat capacity analysis, *Thermochimica Acta*, 2010, **505**, 14–21.
7 J. E. Schawe and K.-U. Hess, The kinetics of the glass transition of silicate glass measured by fast scanning calorimetry, *Thermochimica Acta*, 2019, **677**, 85–90.
8 P. Cebe, X. Hu, D. L. Kaplan, E. Zhuravlev, A. Wurm, D. Arbeiter and C. Schick, Beating the heat--fast scanning melts silk beta sheet crystals, *Sci Rep*, 2013, **3**, 1130.
